# Vitamin C administration in the critically ill: a summary of recent meta-analyses

**DOI:** 10.1186/s13054-019-2538-y

**Published:** 2019-07-30

**Authors:** Anitra C. Carr

**Affiliations:** Department of Pathology and Biomedical Science, University of Otago, P.O. Box 4345, Christchurch, 8140 New Zealand

Since 2018, there has been a dramatic upsurge in publications relating to the use of vitamin C in critically ill patients, particularly those suffering from sepsis [1]. This has primarily been in response to the well-publicized before-and-after study of Marik et al. [2], which indicated that intravenous administration of 6 g/day vitamin C (in combination with thiamine and hydrocortisone) could improve the outcomes of patients with sepsis, including decreasing mortality. Over the past year, seven meta-analyses assessing the effects of vitamin C administration in critically ill patients have been published, with four appearing in the past 4 months alone (see Table 1 summary).

**Table 1:** A summary of recent meta-analysis of vitamin C administration in critical care patients

Publication details	Title	Selection criteria (PICO)	Included studies	Subgroup analysis	Findings
Putzu et al. [3] Crit Care Med	The effect of vitamin C on clinical outcome in critically ill patients: A systematic review with meta-analysis of randomised controlled trials	P—adult critically ill patients I—vitC (any regimen) C—placebo or no therapy O—mortality, acute kidney injury, supraventricular arrhythmia, ventricular arrhythmia, stroke, ICU LOS, hospital LOS	44 RCTs: 16 in ICU setting (*n* = 2857) 28 in cardiac surgery (*n* = 3598)	Mixed ICU vs burns vs sepsis/septic shock vs acute pancreatitis VitC alone vs enteral vitC vs IV vitC vs IV vitC > 5 g	ICU patients: X mortality X acute kidney injury X ICU or hospital LOS Cardiac surgery: ↓ postoperative atrial fibrillation ↓ ICU and hospital LOS
Wang et al. [4] Ann Intensive Care	Effects of different ascorbic acid doses on the mortality of critically ill patients: a meta-analysis	P—critically ill patients I—IV vitC (including co-administration of antioxidants) C—placebo or no intervention O—mortality, resuscitation fluid requirement, urine output, acute kidney injury, vasopressor requirement, duration of mechanical ventilation, ICU and/or hospital LOS	12 RCT, quasi-RCT, observational (*n* = 1210)	Low dose vs medium dose vs high dose Burn vs sepsis vs others	↓ mortality (doses of 3–10 g/day) X morality (< 3 g/day or ≥ 10 g/day) ↓ duration of vasopressor support ↓ duration of mechanical ventilation X acute kidney injury X ICU or hospital LOS X fluid requirement X urine output
Hemila and Chalker [5]	Vitamin C can shorten the length of stay in the ICU: A meta-analysis	P—ICU patients I—vitC C—placebo or none O—ICU LOS, duration of mechanical ventilation	18 controlled trials (*n* = 2004) including 13 cardiac surgery	IV vs oral 1–2 days ICU vs 3–5 days ICU > 24 h ventilation vs < 24 h ventilation	↓ ICU LOS ↓ duration of mechanical ventilation
Langlois et al. [6] JPEN	Vitamin C supplementation in the critically ill: A systematic review and meta-analysis	P—ICU patients I—vitC (enteral or parenteral) C—placebo or none O—mortality, incident infections, ICU LOS, hospital LOS, duration of mechanical ventilation	11 RCTs 9 RCTs with mortality (*n* = 1322)	Low dose vs high dose Combined therapy vs monotherapy Oral/enteral vs parenteral Non-septic vs septic Higher-quality trials vs low-quality trials	X mortality ↓ (trend) mortality (IV high dose vitC monotherapy) X infections X ICU or hospital LOS X duration of mechanical ventilation
Zhang and Jativa [7] SAGE Open Med	Vitamin C supplementation in the critically ill: A systematic review and meta-analysis	P—critically ill adult patients I—IV vitC C—placebo or no intervention O—mortality, duration of mechanical ventilation, duration of vasopressor support, fluid requirements, urine output	4 RCTs and 1 retrospective (*n* = 142)		X mortality ↓ need for vasopressor support ↓ duration of mechanical ventilation ↓ (trend) fluid requirements ↑ (trend) urine output
Li Crit Care [1]	Evidence is stronger than you think: a meta-analysis of vitamin C use in patients with sepsis	P—patients with sepsis I—IV vitC C—placebo or none O—mortality, ICU LOS, vasopressor duration	2 RCTs and 1 before-after		↓ mortality X ICU LOS ↓ vasopressor duration
Lin et al. [8] Open J Intern Med	Adjuvant administration of vitamin C improves mortality of patients with sepsis and septic shock: A systems review and meta-analysis	P—patients with septic shock and severe sepsis I—vitC C—placebo O—mortality	4 RCTs and 2 retrospective studies (*n* = 109)	RCT vs retrospective High dose vs low dose	X mortality ↓ mortality (doses of > 50 mg/kg/day) X ICU LOS

*ICU* intensive care unit, *IV* intravenous, *LOS* length of stay, *PICO* patients, intervention, comparator, outcome, *RCT* randomized controlled trial, *vitC* vitamin C

The most recently published and largest meta-analysis included 44 randomized controlled trials (16 intensive care and 28 cardiac surgery) [9]. Although meta-analyses that include a larger number of studies have increased power, they run the risk of comparing disparate studies. This is particularly the case with vitamin C studies whereby the dose (milligrams vs grams), rout of administration (oral vs intravenous), duration (hours vs days), and disease (e.g., sepsis vs cardiac surgery) can have a large impact on outcomes [10, 11]. Therefore, appropriate subgroup analyses should be carried out, although this is currently challenging due to the low number of comparable studies.

All but one of the current meta-analyses have focused on mortality as a primary outcome, despite many of the included trials having relatively low numbers of patients. In most cases, no effect of intervention was observed on mortality, except in specific subgroup analyses (e.g., sepsis and higher dose intravenous vitamin C). However, there have been few of these studies published to date, and even fewer of high methodological quality [6]. Other commonly assessed outcomes included ICU and hospital length of stay, duration of vasopressor support and mechanical ventilation, and acute kidney injury. Some of the meta-analyses showed decreases in several of these secondary outcomes, while others showed no effect, depending on the selection criteria used for study inclusion (Table 1).

There are currently over a dozen randomized controlled trials registered on clinicaltrials.gov that are assessing the effects of vitamin C administration in critically ill patients, particularly those with sepsis. One would hope that in the short term, no more meta-analyses appear every time another small study is published, but instead wait until some of the larger trials (such as VICTAS and LOVIT) have been completed. Otherwise, there may end up with more meta-analyses than published trials.

## Data Availability

NA
